# Impact of Glow-Discharge Nitriding Technology on the Properties of 3D-Printed Grade 2 Titanium Alloy

**DOI:** 10.3390/ma17184592

**Published:** 2024-09-19

**Authors:** Janusz Kamiński, Ryszard Sitek, Bogusława Adamczyk-Cieślak, Krzysztof Kulikowski

**Affiliations:** Faculty of Materials Science and Engineering, Warsaw University of Technology, Wołoska 141, 02-507 Warsaw, Poland; ryszard.sitek@pw.edu.pl (R.S.); boguslawa.cieslak@pw.edu.pl (B.A.-C.); krzysztof.kulikowski@pw.edu.pl (K.K.)

**Keywords:** titanium Grade 2, DMLS, corrosion, EIS, microhardness profile, glow-discharge nitriding

## Abstract

This study presents a comparative analysis of the corrosion resistance of nitrided layers on conventional Grade 2 titanium alloy and those produced by direct metal laser sintering (DMLS). Low-temperature glow-discharge nitriding of the tested materials was carried out using conventional glow-discharge nitriding (so-called nitriding at the cathode potential—TiN/CP) and with the use of an “active screen” (nitriding at the plasma potential—TiN/PP). The TiN + Ti_2_N + Ti(N) layers were characterized by their microstructure, nanohardness profile distribution, surface topography, and corrosion resistance. The reduction in the cathodic sputtering phenomenon in the process using the active screen allowed the creation of surface layers that retained the topography of the base material. The parameters of the glow-discharge treatment led to grain growth in the printed substrates. This did not adversely affect corrosion resistance. The corrosion resistance of nitrided layers on the printed titanium alloy is only slightly lower than that of layers on the conventional Grade 2 alloy. Iron precipitates at grain boundaries facilitate increased nitrogen diffusion, resulting in reduced nitrogen concentration in the surface layer, slight changes in corrosion potential values, and increased nitrogen concentration in the Ti(N) diffusion layer.

## 1. Introduction

Titanium and its alloys have been extensively studied since the early 1950s due to their broad range of applications across various industries, including aviation, space, shipbuilding, chemical, defense, construction, and medicine industries [1,2,3,4,5,6,7,8,9,10]. The versatility of titanium alloys stems from their ability to be tailored through variations in chemical composition and phase structure [11].

The Grade 2 titanium alloy, widely used in medical applications, offers high corrosion resistance, reduced density, high specific strength, low Young’s modulus, and excellent biocompatibility [10,12]. However, its applications are limited by its low wear resistance, resulting from low hardness and susceptibility to flaking and galling at frictional contact points [13]. This is particularly crucial in medical devices, such as orthopedic implants, where these limitations can lead to the release of alloy components into surrounding tissues, causing inflammatory responses and osteolysis [14]. To enhance the service properties of titanium alloys, various surface engineering techniques have been developed [15], including the application of glow-discharge nitriding to create surface layers [16].

In conventional glow-discharge nitriding, conducted at the cathode potential, cathodic sputtering removes oxides and adsorbed gases from the surface layer, creating numerous structural defects that facilitate the formation of nitrided layers. However, this process can result in preferential sputtering, overheating of sharp edges, and increased surface roughness, leading to non-uniformity. These effects can limit the suitability of conventionally nitrided titanium for advanced medical applications, such as cardiovascular implants [17]. Consequently, conventional glow-discharge nitriding is primarily used to enhance the durability and reliability of titanium and its alloys, various grades of steel, including austenitic steel, and nickel alloys in industrial applications [18,19,20].

To mitigate the adverse effects associated with conventional nitriding, particularly the edge effect, glow-discharge nitriding can be performed using an active screen operated at the plasma potential. This modification produces titanium nitride layers characterized by a homogeneous structure across the entire surface, including edges, while preserving the base material’s original topography. This technique is particularly advantageous for small-sized components where surface integrity is critical [21].

The fundamental difference between conventional glow-discharge nitriding (cathode potential, CP) and active screen nitriding (plasma potential, PP) lies in the heating method. In conventional nitriding, heating occurs through radiation from near-cathode glow-discharge regions and ion bombardment, while in active screen nitriding, heating is achieved by convection from the active screen and the presence of non-equilibrium and non-isothermal plasma. Regardless of the method, both techniques create TiN + Ti_2_N + αTi(N) diffusion layers with nanostructured titanium nitride (TiN) on the surface, exhibiting excellent resistance to abrasive wear, high hardness, corrosion resistance, and biocompatibility. The thickness of the individual layers is primarily determined by the parameters of the glow treatment, including temperature, time, and the chemical composition of the reactive atmosphere, as well as the substrate’s chemical composition [22,23].

Corrosion studies of nitrided layers produced under glow-discharge conditions [16,17] have shown that, apart from temperature, which is one of the main factors influencing changes in roughness [24], there is also the presence or absence of an active screen. Its use influences not only corrosion resistance and surface topography but also the biological activity of the substrate [25].

Recent advancements in nitriding technology have extended to materials produced via additive manufacturing methods. These methods enable the rapid fabrication of complex geometries without the need for traditional machining but introduce anisotropy in mechanical and electrochemical properties depending on the analyzed plane [26]. The necessity of nitriding at temperatures exceeding 500 °C to achieve efficient nitrogen diffusion in titanium can induce martensitic transformation in the core, resulting in increased grain size [27]. However, research by Kaminski [28] demonstrated the feasibility of forming TiN layers on NiTi alloys at approximately 300 °C. These layers, produced by conventional glow-discharge nitriding, were characterized by limited thickness, increased roughness, and electrochemical heterogeneity, rendering the substrates more susceptible to pitting corrosion and limiting their applicability in medical contexts.

Although substantial research exists on the nitriding of titanium and its alloys [29,30], as well as on the use of 3D printing techniques for producing titanium components [31], there is limited knowledge regarding surface treatments for 3D-printed titanium substrates. Therefore, this study aims to evaluate the effects of conventional glow-discharge nitriding (cathode potential) and active screen nitriding (plasma potential) on the microstructure and corrosion resistance of Grade 2 titanium produced using direct metal laser sintering (DMLS) compared to conventional Grade 2 titanium alloy.

## 2. Materials and Methods

### 2.1. Specimen Preparation

The material tested was Grade 2 titanium with the following chemical composition: Fe ≤ 0.30%, C < 0.1%, H ≤ 0.015%, N ≤ 0.03%, O ≤ 0.18%, and the remainder Ti. For the conventional Grade 2 alloy, samples were cut from a rod with a diameter of 20 mm, while the printed samples were fabricated as cuboids with dimensions of 20 × 15 × 10 mm using a 3D printer. An EOS M100 (Krailling, Germany) working with DMLS (direct metal laser sintering) technology using Grade 2 spherical powder was used. The cuboids were then cut into samples measuring 20 × 15 × 2 mm. The nominal diameter of a spherical powder particle ranges from 15 to 45 µm. In our recent work [20], we investigated the particle size and sphericity of Grade 2 powder and described the sample construction process. Later in the article, printed samples were called DMLS, while the conventional titanium alloy was called Grade 2.

### 2.2. Glow Nitriding Process

**Figure 1:**
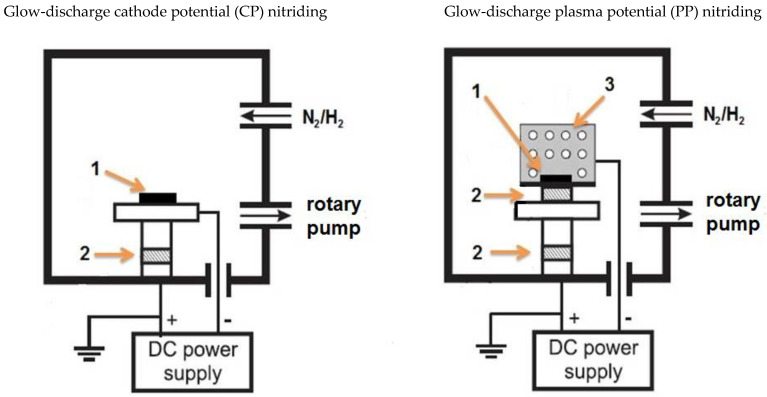
Diagram of devices used in glow-discharge treatments: 1—sample, 2—electrical insulator, 3—active screen made of perforated titanium sheet.

Before the glow nitriding process, the surfaces of all samples (conventional Grade 2 titanium alloy and a specimen built with DMLS in the x-z plane) were ground on sandpaper at increasing grit to 1200’, then washed with acetone in an ultrasonic cleaner. Both the glow-discharge assisted processes (at the cathode potential (denoted as TiN/CP) and at the plasma potential (denoted as TiN/PP)) used the same technological parameters of T = 680 °C, process time t = 6 h, and a pressure of p = 1.9 hPa in a reactive atmosphere consisting of 95% N_2_ + 5% H_2_. The presence of hydrogen in the reactive atmosphere resulting from the formation of atomic hydrogen under glow-discharge conditions facilitates the removal of the surface layer of titanium oxides formed spontaneously during the preparation of the test samples [17]. Figure 1 shows the schematic appearance of the glow treatment chamber processed at the plasma potential and cathode potential. The main difference between the technologies used is the presence of an active screen, which, on the one hand, protects the samples against direct glow discharge (defecting the structure), but requires a much higher power consumption to obtain the set temperature. This makes processes carried out at the plasma potential more costly from an economic point of view.

The surface topography of the nitrided layers was measured by means of an ACCURION optical profilometer, HALCYONICS_i4 Sensofar Metrology (Barcelona, Spain), using the SensoVIEW program (1.8.0). The initial surface and nitrided layers were observed using the Scanning Electron Microscope (SEM) HITACHI SU8000 (Hitachi High-Tech, Tokyo, Japan) equipped with adapters for microanalysis in microareas (EDS—Energy Dispersive Spectroscopy). Nitrided layers were observed on the surface (longitudinal) (in secondary electron (SE) mode) and in cross-section (transversal) (electron backscatter diffraction EBSD mode) after chemical etching in Kroll solution (2 mL HNO_3_ + 2 mL HF + 96 mL H_2_O).

### 2.3. Nanohardness of the Nitrided Specimen

Nanoindentation tests were performed on polished DMLS (TiN/CP and TiN/PP) and Grade 2 (TiN/CP and TiN/PP) specimens using a Berkovich indenter on NanoTest Vantage Alpha by Micro Materials, Ltd. (Wrexham, UK). The determined load–displacement curves as a function of the indenter displacement were analyzed according to the Oliver–Pharr method to evaluate nanohardness. A total of 144 indentations were carried out across the nitrided layer and substrate in zig-zag displacement with a 0.3 µm distance, 1 mN load, 10 s loading time, 10 s unloading time, and 5 s dwell period.

### 2.4. XRD Diffraction Test

The phase composition of the samples was studied using the X-ray diffraction (XRD) method. The phase composition was identified using a Bruker D8 Discover diffractometer (Bruker AXS, Karlsruhe, Germany) using a copper point focus X-ray tube, while the radiation length of CuKα was λ = 0.154056 nm. Data were collected from 10° to 130°, with a step size of 0.025° and a count time of 10 s per step. X-ray diffraction (XRD) patterns were analyzed using Bruker EVA software (V3.0) and the PDF-2 database (from the International Centre for Diffraction Data).

### 2.5. Corrosion Resistance Test

Corrosion resistance examinations were carried out by means of the impedance and the potentiodynamic methods using an Autolab PGSTAT100 potentiostat/galvanostat (Eco Chemie B.V., Utrecht, The Netherlands) with an FRA2 module, in non-deaerated Ringers solution (NaCl—8.6 g/dm^3^, KCl—0.3 g/dm^3^, CaCl_2_ × 2H_2_O—0.33 g/dm^3^) at 37 °C. Prior to electrochemical tests, the samples were exposed to the Ringer solution in current-free conditions for 3600 s. Impedance examination was conducted in a three-electrode setup—the test electrode, reference electrode (saturated calomel electrode (SCE)), and auxiliary electrode (platinum)—with the frequency ranging from 10^5^ Hz to 10^−3^ Hz and use of an AC signal with an amplitude of 10 mV. The corrosion cell was kept in a Faraday cage. EIS methods were recorded in the potentiostatic mode at open circuit potential (EOCP). Eco Chemie Analyst software [EQUIVRT—Baukamp program (4.9.007)] was used for processing and fitting the impedance spectra. An equivalent circuit with two time constants R(Q[R(RQ)]) [32] was used to analyze the spectra of nitrided layers, indicating high electrochemical heterogeneity of the substrate, especially in the case of the nitrided layers produced at the cathode potential, characterized by significant surface development, where the conditions of diffusion significantly influence the electrochemical parameters of the layer. The obtained spectra are presented in the form of a Bode plot.

Potentiodynamic tests were conducted in an identical trielectrode setup, up to a potential of 3000 mV. Polarization of the tested material was performed with a rate of 0.2 mV/s. The corrosion potential and current density were determined from the Tafel plots.

## 3. Results and Discussion

**Figure 2:**
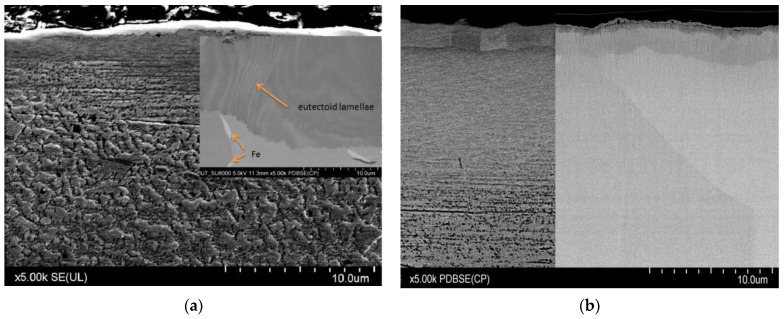
Microstructures of the nitrided layers DMLS-TiN/PP (**a**) and DMLS-TiN/CP (**b**) produced on a printed Grade 2 titanium alloy in the etched and unetched states with Kroll’s reagent. Inset: the distribution of iron and eutectoid lamellae in the Ti_2_N layer is marked with arrows.

Figure 2 presents the microstructures of the TiN/PP and TiN/CP layers, produced on a substrate via direct metal laser sintering (DMLS). Both microstructures exhibit the typical multiphase nature of nitrided layers, characterized by TiN + Ti_2_N + Ti(N). The thickness of the TiN and Ti_2_N phases in these samples appears comparable, but variations in the thickness of the diffusion Ti(N) layer are observed, which may be attributed to differences in nitrogen particle kinetics. The enhanced nitrogen diffusion observed in the TiN/CP layers could be due to the specific substrate characteristics.

The presence of eutectoid plates in the nitrided layers [31] is likely due to the significant thermal gradient resulting from the glow nitriding process during cooling. Additionally, the microstructural analysis of DMLS-TiN/CP layers reveals a rapid transition in properties from the surface to the substrate, which may limit their long-term operation due to the formation of a hard and brittle layer on the surface with reduced plasticity [27], alongside the considerable stress distribution changes [33].

**Figure 3:**
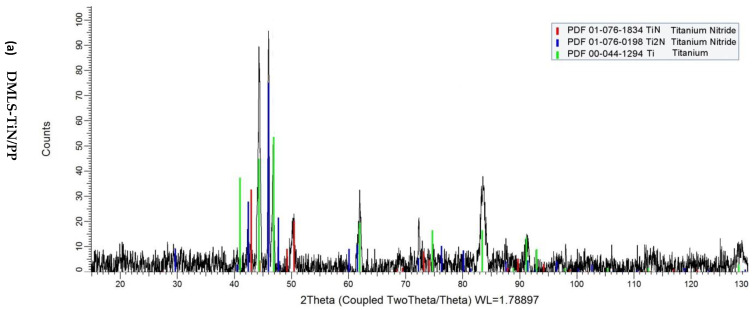

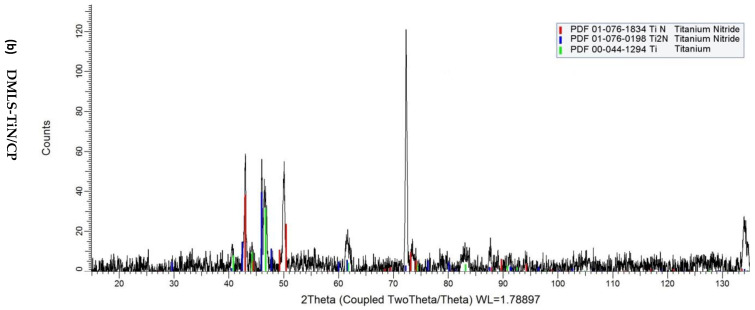
Phase compositions of the nitrided layers DMLS-TiN/PP (**a**) and DMLS-TiN/CP (**b**) produced on a printed Grade 2 titanium alloy.

The XRD analysis of nitrided layers produced on 3D-printed Grade 2 titanium alloy using conventional (CP) and active screen (PP) nitriding is shown in Figure 3. In both cases, the phase composition is identical (TiN + Ti_2_N + Ti(N)), though differences in peak intensities reflect variations in phase thickness. A pronounced peak around 72° in the TiN/CP layer suggests strong texturing of the Ti_2_N phase. This observation provides insights into the anisotropic properties of the printed substrates [34].

**Figure 4:**
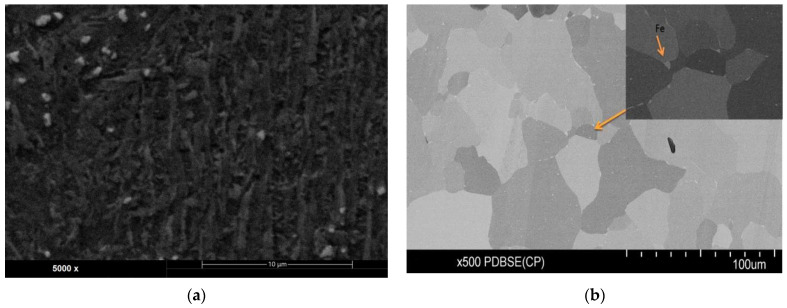
Microstructure of the printed (DMLS) Grade 2 alloy in the initial state (**a**) and post-glow-discharge treatment (**b**). Inset: the distribution of iron.

Figure 4 shows the microstructure of the Grade 2 alloy printed (using DMLS technology) in the XZ plane in the initial state (Figure 4a) and post-glow-discharge treatment (Figure 4b).

In the case of the starting material (Figure 4a), a fine-grained structure composed of α’ martensitic lamellae is observed. The applied glow treatment parameters (in particular the process temperature of 680 °C) resulted in a significant increase in the grain size in Figure 4b. The observed brighter spots on the grain boundaries indicate the segregation of iron along the grain boundaries. The addition of iron to Grade 2 powder enhances the stabilization of the martensitic phase in the starting material [27], but it also increases nitrogen diffusion across the grain boundaries [35] during the glow nitriding process.

**Figure 5:**
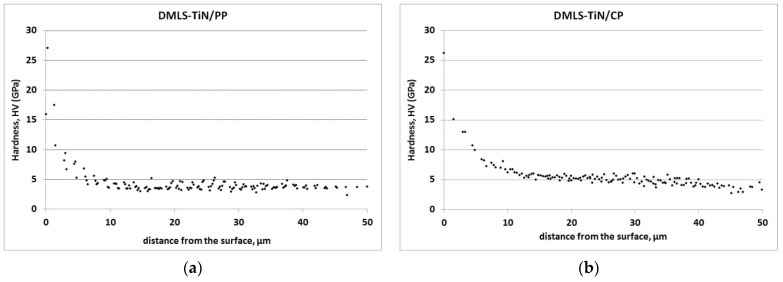
Nanohardness as a function of depth measured along cross-sections of the nitrided (TiN/PP (**a**) and TiN/CP (**b**)) DMLS specimens.

**Figure 6:**
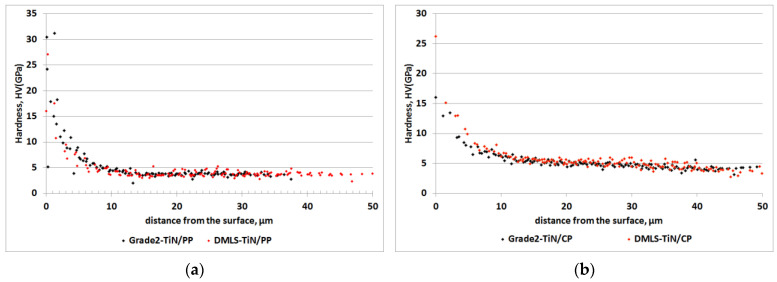
Nanohardness as a function of depth measured along cross-sections of the nitrided (TiN/PP (**a**) and TiN/CP (**b**)) DMLS and Grade 2 specimens.

The hardness distribution of the nitrided layers, as shown in Figure 5, demonstrates that TiN/CP layers exhibit a ~20% increase in hardness compared to TiN/PP layers. Additionally, when comparing the hardness distribution of nitrided TiN/CP layers depending on the structure of the substrate (DMLS and Grade 2) (Figure 6a), a slight (approx. 4%) increase in its value was found in the diffusive Ti(N) layer in the DMLS substrate compared to the conventional one. These results indicate that an increased number of structural defects or segregation of iron along grain boundaries facilitates the diffusion of nitrogen into the substrate, favoring, among other things, an increase in its hardness at a further distance from the surface. In the case of nitrided TiN/PP layers, where nitrogen particles are characterized by lower kinetics, no influence of the substrate structure (DMLS and Grade 2) on the microhardness distribution was observed (Figure 6b).

**Figure 7:**
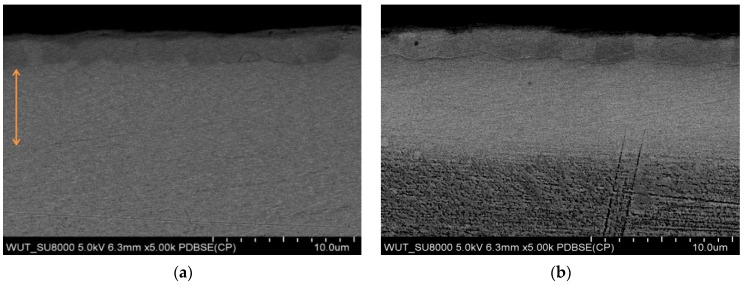
Microstructure of the nitrided layer (TiN/CP) produced on a printed titanium alloy (DMLS) (**b**) compared to the conventional Grade 2 titanium alloy (**a**). Inset: orange arrow—the extent of the diffusive Ti(N) layer in the Grade 2 titanium alloy.

By analyzing the hardness graph in Figure 5, the thicknesses of the nitrided TiN + Ti_2_N + Ti(N) layer on the printed Grade 2 titanium alloy can be estimated: approximately 15 µm for DMLS-TiN/CP and 8 µm for DMLS-TiN/PP. The data are consistent with hardness measurements for individual titanium nitrides (TixNy) [36]. Further comparisons (Figure 7) reveal that the Ti(N) diffusion layer in printed substrates is more pronounced due to increased nitrogen concentration following 3D printing.

In the case of the nitrided layer produced using an active screen on the conventional Grade 2 alloy, due to the low-temperature glow nitriding (680 °C), the thickness of the TiN/Ti_2_N layer was insignificant and amounted to approximately 1 µm. Similar values were obtained by Tarnowski [17] in his research.

**Figure 8:**
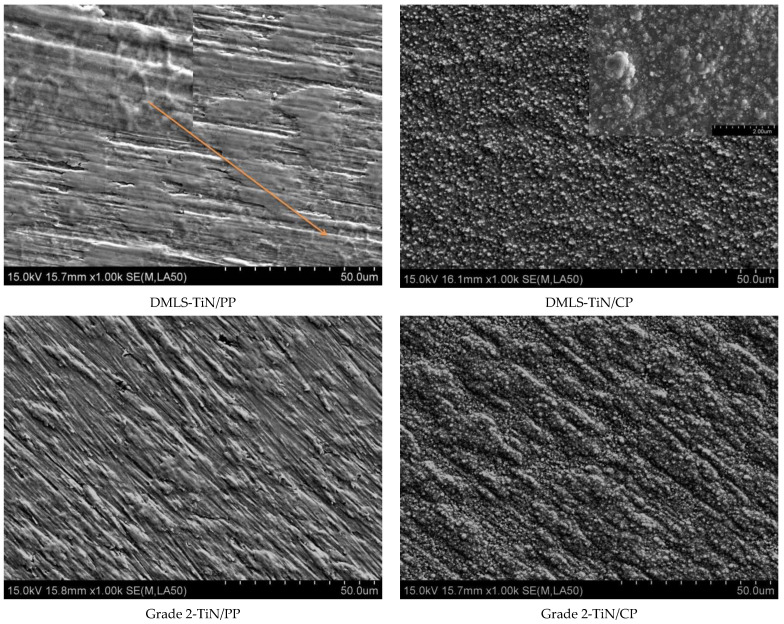
Topographies of layers created at the plasma (TiN/PP) and cathode potentials (TiN/CP) on printed (DMLS) and conventional (Grade 2) substrates.

Figure 8 shows the surface topographies of the produced layers, while Table 1 lists the characteristic values of their roughness.

The surface topographies of the nitrided layers produced on DMLS and conventional Grade 2 substrates (Figure 8) reveal that layers created via an active screen closely follow the topography of the initial material. In contrast, those produced at the cathode potential exhibit a combination of equiaxed and thicker blocky crystallites. The share of blocky crystallites correlates with processing temperatures [30], influencing surface roughness.

Table 1 summarizes the results of testing the roughness of nitrided layers produced with two types of glow-discharge nitriding (at cathode potential (TiN/CP) and plasma potential (TiN/PP)) on two types of substrates (conventional Grade 2 and DMLS) compared to the initial material (IS).

**Figure 9:**
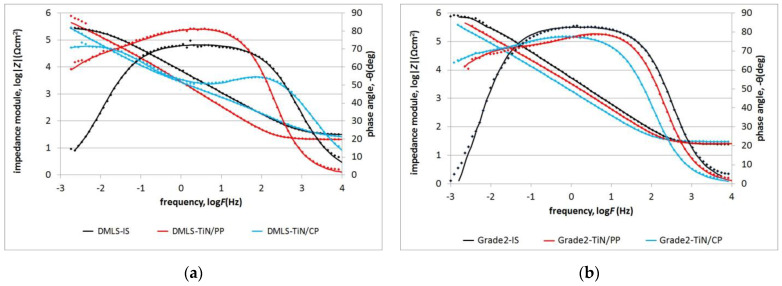
Bode plots of nitrided layers produced at plasma (TiN/PP) or cathode potential (TiN/CP) on printed (**a**) and classic (**b**) substrates compared to the starting materials (IS). Date—dotted line; fit—solid line.

The analysis of the data presented in Table 1 indicates that the low-temperature glow nitriding process using an active screen (TiN/PP) does not affect changes in roughness, regardless of the initial structure of the substrate. This result confirms previous literature reports [25] that the obtained layers reflect the topographies of the initial material, which favors their wide use in medical applications. In the case of classic glow-discharge nitriding (at the cathode potential), an increase in roughness is observed in both analyzed cases. However, significant differences in the obtained values can be noticed; in the case of the DMLS substrate, an approximately 25% increase in roughness parameters is observed, significantly exceeding the values obtained in the classic Grade 2 alloy. The obtained result is influenced by both the growth of numerous grains under the influence of temperature [37] and the formation of blocky crystallites of titanium nitrides [30]. The obtained layer roughness data translate into the results of impedance tests (Figure 9a), in which the electrochemical parameters of the dielectric layer (DMLS TiN/CP) indicate their diffusive nature.

Figure 9 shows the impedance spectra of the nitrided layers (compared to the initial materials (IS)) produced on a printed substrate (DMLS) compared to nitrided layers produced on the conventional Grade 2 titanium alloy.

The electrochemical parameters of nitrided layers produced in low-temperature plasma increase the corrosion resistivity of the substrate, regardless of the structure of the initial material (Table 2). At the same time, the shape of the impedance spectra indicates a clear increase in the electrochemical heterogeneity of the layer, as evidenced by the presence of additional capacitive peaks. In the case of layers created at the plasma potential, due to the fact that they do not change the roughness, a significant share of diffusion factors resulting from the roughness of the initial material (Ra = 0.2 µm) is observed in the layer, as evidenced by the value of the n parameter’s (n = 0.60–0.67) double layer. For the TiN layer produced on the printed DMLS-TiN/PP titanium alloy, both an increased phase angle value and a higher phase angle at low frequencies are observed, which enhance its corrosion resistance in the tested environment. 

Significant differences in the quality of the dielectric layer are observed in the case of nitrided TiN/CP layers produced on titanium alloys with different structures.

In the case of nitrided layers produced on a DMLS substrate, the layer has a diffusive character (n = 0.63) with a significant surface area dominated by diffusion processes, while on the conventional Grade 2 titanium alloy, the dielectric layer has a slightly reduced capacitive character (n = 0.87). This result indicates that the parameters of titanium glow treatment—especially the classic one nitrided at the cathode potential—cannot be implemented in the case of printed substrates. EIS studies conducted on Grade 2 titanium alloy showed that the produced nitrided layers TiN/PP and TiN/CP exhibit similar phase angle values; however, the TiN/PP sample demonstrated a higher impedance modulus at low frequencies, which translates to an observed increase in its corrosion resistance. In both cases, an increase in the Rt resistance value (Table 2) of the nitrided layers is observed compared to the reference sample.

**Figure 10:**
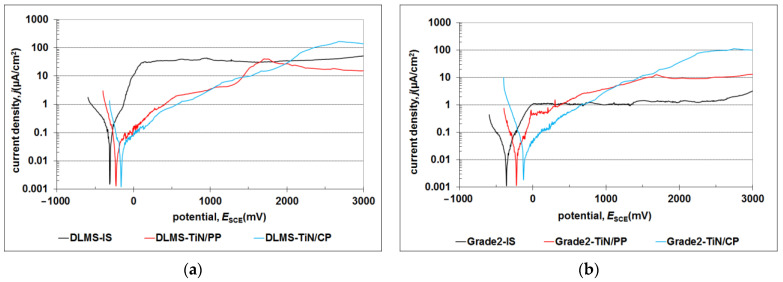
Potentiodynamic curves of glow-discharge layers formed on titanium Grade 2 (DMLS (**a**) and conventional (**b**)) exposed in a Ringer’s solution at 37 °C.

The conducted potentiodynamic tests, the results of which are presented in Figure 10 and Table 3, confirm the increase in the corrosion resistance of the nitrided layers produced on the printed titanium alloy compared to the starting material (DMLS IS). This result was not confirmed for the classic Grade 2 alloy; in this case, TiN/CP layers are characterized by slightly reduced corrosion resistance compared to the starting material. The obtained result is consistent with the literature data [16,17] indicating a decrease in corrosion resistance in the following order, TiN/PP > IS > TiN/CP, in the classic Grade 2 titanium alloy.

**Figure 11:**
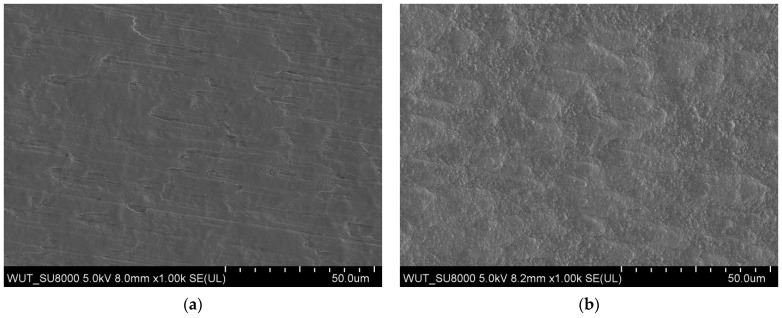
Topography of the nitrided TiN/PP (**a**) and TiN/CP (**b**) layers produced on the 3D-printed Grade 2 titanium alloy after corrosion tests in Ringer’s solution at 37 °C.

The shift of the corrosion potential values towards the anodic side indicates the cathodic nature of the TiN layers. No local corrosion initiation was observed in the entire range of tested potentials (Figure 11).

The observed change in the value of the corrosion potential is determined by both the presence of nitrogen in the surface layer and its susceptibility to oxidation to form a mixture of TiNxOy oxides [38], as well as the increased roughness of the layer created at the cathode potential, increasing the active surface of the nanometric oxide layer. The estimated nitrogen concentration in the surface layer (EDS method) showed an increase in nitrogen concentration in the TiN/CP layer compared to layers produced using an active screen (TiN/PP). The increased nitrogen saturation of the TiN/CP surface layer results from the greater kinetic energy of the particles present in conventional glow-discharge nitriding. However, by comparing the values of the corrosion potentials of the tested layers depending on the structure of the starting material, differences can be found in the influence of nitrogen concentration on the potential value. In the case of the classic Grade 2 alloy, relatively high nitrogen concentrations are observed in the TiN/CP (approx. 35% at. N) and TiN/PP (approx. 25% at. N) layers, which translates into a significant shift in the values of corrosion potentials, respectively by ∆*E*_corr_ = +140 mV(TiN/PP) and ∆*E*_corr_ = +240 mV(TiN/CP). In the case of a printed substrate, despite identical glow-discharge treatment parameters, nitrogen concentrations in the layers are significantly lower—approximately 26% at. N (TiN/CP) and 22% at. N (TiN/PP). This result means that there are no significant changes in the values of corrosion potentials (∆*E*_corr_ = +80 mV(PP) and ∆*E*_corr_ = +140 mV(CP)), as well as no significant differences in the surface hardnesses of both substrates (Figure 5). It can be assumed that the obtained result is determined by the structure of the DMLS substrate, in which there are numerous easy diffusion paths (grain boundaries), which facilitate easier penetration of nitrogen into the substrate, as well as the presence of iron precipitates located mainly on the grain boundaries, which also favor nitrogen diffusion [35]. Both of these factors cause an increase in the nitrogen concentration in the Ti(N) diffusion layer, at the expense of a reduced nitrogen concentration in the nitrided TiN/Ti_2_N layer.

## 4. Conclusions

The microstructure analysis showed that the nitrided layers on both types of titanium alloy consisted of TiN, Ti_2_N, and Ti(N) phases. The layers produced by the traditional nitriding process (TiN/CP) had a rougher surface compared to those produced using the active screen (TiN/PP). This difference is attributed to the reduced cathodic sputtering in the TiN/PP process, which results in less surface damage and better preservation of the base material’s topography. Corrosion resistance tests revealed that both nitrided layers improved the corrosion resistance of the titanium alloy compared to the untreated direct metal laser sintering (DMLS) material. The TiN/CP layers, however, showed slightly lower corrosion resistance than the TiN/PP layers. This difference is attributed to the more uniform microstructure of the TiN/PP layers, which may reduce the number of potential sites for corrosion initiation. This study demonstrates that nitriding of Grade 2 titanium alloy produced by direct metal laser sintering slightly reduced the corrosion resistance compared to traditional titanium Grade 2 alloys. The nanohardness measurements indicated that the surface layers had significantly higher hardness compared to the substrate material. The hardness decreased gradually from the surface towards the interior, which is typical for nitrided layers. The TiN/PP layers exhibited slightly lower hardness compared to the TiN/CP layers, which is likely due to the more homogeneous microstructure and the absence of the preferential sputtering effect.

## Figures and Tables

**Table 1:** Surface roughness parameters of nitrided (TiN/CP and TiN/PP) layers in comparison to materials (DMLS and Grade 2) in initial state (IS).

	Sa (µm)	SD	Sp (µm)	SD	Sv (µm)	SD	Sz (µm)	SD
DMLS-IS	0.221	0.012	1.053	0.021	1.300	0.068	2.354	0.081
DMLS-TiN/PP	0.224	0.005	1.110	0.026	1.279	0.043	2.390	0.039
DMLS-TiN/CP	0.275	0.018	1.371	0.079	1.554	0.085	2.926	0.162
Grade 2-IS	0.197	0.01	1.015	0.035	1.020	0.079	2.035	0.055
Grade 2-TiN/PP	0.198	0.006	1.008	0.056	1.059	0.039	2.068	0.068
Grade 2-TiN/CP	0.234	0.006	1.124	0.022	1.311	0.069	2.435	0.078

Sa—arithmetic mean of the surface deviation from the average surface; Sp—height of the highest elevation of the surface; Sv—depth of the lowest surface depression; Sz—max surface height (Sp + Sv); SD—sample standard deviation.

**Table 2:** Characteristic electrochemical parameters of the nitrided layers (impedance tests).

			Dielectric Layer	Error (%)	Double Layer	Error (%)
DMLS-IS	R (Ωcm^2^)				2.98 × 10^5^	0.8
	Q_CPE_	Y_0CPE_(Fcm^−2^s^n−1^)			3.00 × 10^−5^	0.3
		n			0.82	0.1
DMLS TiN/PP	R (Ωcm^2^)		5.99 × 10^4^	11.7	2.92 × 10^6^	35.9
	Q_CPE_	Y_0CPE_(Fcm^−2^s^n−1^)	6.29 × 10^−5^	0.5	1.77 × 10^−5^	6.0
		n	0.92	0.1	0.60	4.2
DMLS TiN/CP	R (Ωcm^2^)		2.57 × 10^3^	7.2	7.36 × 10^6^	45.5
	Q_CPE_	Y_0CPE_(Fcm^−2^s^n−1^)	1.66 × 10^−4^	4.7	8.42 × 10^−5^	17.8
		n	0.63	2.8	0.81	4.2
Grade 2 IS	R (Ωcm^2^)				9.44 × 10^5^	1.8
	Q_CPE_	Y_0CPE_(Fcm^−2^s^n−1^)			2.75 × 10^−5^	0.4
		n			0.92	0.1
Grade 2 TiN/PP	R (Ωcm^2^)		2.74 × 10^4^	15.1	2.46 × 10^6^	18.4
	Q_CPE_	Y_0CPE_(Fcm^−2^s^n−1^)	5.40 × 10^−5^	1.2	1.92 × 10^−5^	3.9
		n	0.91	0.2	0.67	2.5
Grade 2 TiN/CP	R (Ωcm^2^)		1.56 × 10^5^	8.2	2.43 × 10^6^	11.9
	Q_CPE_	Y_0CPE_(Fcm^−2^s^n−1^)	1.07 × 10^−4^	0.3	2.36 × 10^−5^	8.9
		n	0.87	0.1	0.69	3.4

R—resistance; Q_CPE_—capacity of the constant phase element; Y_0CPE_—admittance; n—coefficient of imperfections of the constant phase element (CPE), an empirical constant ranging from 0 to 1. It is worth noting that when n = 1, the CPE behaves as a pure capacitor, while when n = 0, the CPE behaves as a pure resistor.

**Table 3:** Characteristic electrochemical parameters of the nitrided layers.

	DMLS			Conventional Grade 2		
	*R_p_* (kΩcm^2^)	*E*_corr (SCE)_ (mV)	*I*_corr_ (µA cm^−2^)	*R_p_* (kΩcm^2^)	*E*_corr (SCE)_ (mV)	*I*_corr_ (µA cm^−2^)
TiN/CP	1122	−165	2.65 × 10^−2^	792	−125	2.53 × 10^−2^
TiN/PP	1194	−230	1.90 × 10^−2^	1603	−225	1.42 × 10^−2^
IS	275	−310	7.13 × 10^−2^	1369	−360	1.91 × 10^−2^

*E*_corr_—corrosion potential, *I*_corr_—corrosion current density, *R_p_*—polarization resistance.

## Data Availability

The original contributions presented in the study are included in the article, further inquiries can be directed to the corresponding author.
